# Does the use of an intramedullary nail alter the duration of external fixation and rate of consolidation in tibial lengthening procedures? A systematic review

**DOI:** 10.1007/s11751-012-0144-5

**Published:** 2012-10-19

**Authors:** S. Jain, P. Harwood

**Affiliations:** Department of Trauma and Orthopaedic Surgery, Leeds General Infirmary, Great George Street, Leeds, LS1 3EX UK

**Keywords:** Ilizarov, Tibia, Lengthening, Nail, Intramedullary

## Abstract

We performed this systematic review to evaluate tibial lengthening procedures with the use of an intramedullary nail. We investigated the hypothesis that lengthening over a nail can reduce the time spent in an external fixator and increase the rate of consolidation thereby reducing the risk of complications and improving patient satisfaction. We conducted a comprehensive literature search using the MEDLINE, EMBASE and PubMed databases using the key words ‘tibia’ or ‘tibial lengthening’ and ‘nail’. This search was performed in December 2011 and repeated by both authors. Specific outcome measures were the duration of external fixation, rate of consolidation and complication rates. A total of 6 comparative studies published between 2005 and 2011 consisting of 494 procedures met our inclusion and exclusion criteria and were eligible for critical appraisal. The methodological quality of the studies was variable, and they were not homogenous enough for meta-analysis. Patients who have tibial lengthening over an intramedullary nail spend significantly less time in an external fixator. However, there is no reliable evidence to suggest that the rates of consolidation or complication are any different to those lengthened without an intramedullary nail.

## Introduction

Distraction osteogenesis is a widely used technique for limb lengthening [1]. After corticotomy, the applied external fixator enables gradual distraction to achieve the desired length after which follows a consolidation phase for the regenerate column to mature.

The prolonged use of external fixation, necessary in this technique, is associated with numerous complications including pin site infection which has a prevalence of up to 80 % [2]. Other adverse events associated with sustained external fixation when used in distraction osteogenesis include contractures, joint subluxation, axial deviation, late bowing, refracture, pain and sleep disturbance [3].

In response to this problem, the technique of lengthening over intramedullary nails has emerged. Paley described this technique first in the femur and concluded that it was associated with a decrease in the duration of external fixation, protection against refracture and facilitated earlier rehabilitation [4]. This technique has gained wide acceptance because it offers considerable improvement in patient comfort [5], leading to considerable work into tibial lengthening procedures [6]. Despite the perceived advantages, there have also been reports regarding complications associated with this technique, for example, slow consolidation of the regenerate, metalwork failure [7] and deep infection [8].

To evaluate whether tibial lengthening with an intramedullary nail alters the duration of external fixation and rate of consolidation, we reviewed studies that compared these outcome measures against patients lengthened without an intramedullary nail. Complications associated with this technique were also examined, with particular emphasis to pin site infection, deep infection and the need for further surgical intervention.

## Methods

A comprehensive literature search was performed on 7 December, 2011 using MEDLINE^®^ and in 1946–2011 using OVID . Twenty-three hits were obtained using Boolean search methods on ‘tibia lengthening or tibial lengthening’ and ‘nail’.

Studies were selected based on the following eligibility criteria after review of the abstracts:

- Comparative studies where tibial lengthening was performed over an intramedullary tibial nail and compared to tibial lengthening without the use of an intramedullary tibial nail. No language, publication date or publication status restrictions were applied.
- All patients undergoing tibial lengthening were included.
- The Ilizarov method of lengthening was used with a circular or hybrid external fixation device either with or without an intramedullary tibial nail.
- The external fixation index and the consolidation index were reported. These were the primary outcome measures.
- Complications were reported. These were the secondary outcome measures.

**Fig. 1:**
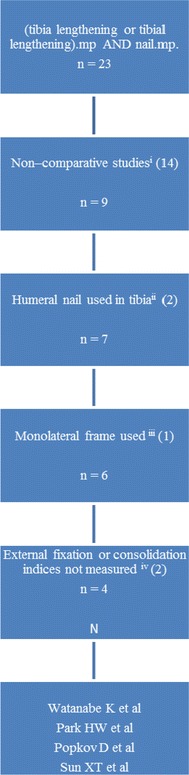
Flow chart showing results of Medline search and application of inclusion and exclusion criteria. *i* Bonnevialle P et al., Chen CM et al., Huang SC et al., Kenawey M et al., Kim H et al., Kim SJ et al., Krieg AH et al., Liu B et al., Schiedel FM et al., Sulaiman et al., Song HR et al., Xia HT et al., Zhao L et al. (search revealed same paper twice). *ii* Chen D et al., Chen D et al. *iii* Chen D et al. *iv* Shyam AK et al., Huang SC et al

Studies that did not fit the above criteria such as use of humeral nails in the tibia, monolateral fixators, non-comparative and duplicate studies were excluded. It was essential for the studies to have compared the results between the study groups and presented these differences with statistical analysis. A flow chart is presented in Fig. 1 showing how the following four papers were selected for review.

- Tibial lengthening over an intramedullary nail by Watanabe et al. [9].
- Tibial lengthening over an intramedullary nail with use of the Ilizarov external fixator for idiopathic short stature by Park et al. [10].
- Flexible intramedullary nail use in limb lengthening by Popkov et al. [11].
- Complications and outcome of tibial lengthening using the Ilizarov method with or without a supplementary intramedullary nail by Sun et al. [12].

A further search using the same terms from EMBASE^®^ (1947–2011) and Pubmed^®^ search engines was performed. The EMBASE^®^ search revealed no further suitable studies, but the Pubmed^®^ search revealed two more studies suitable for review.

- Comparative study of callus progression in limb lengthening with or without intramedullary nail with reference to the pixel value ratio and the Ru Li’s classification by Sun et al. [13].
- Tibial lengthening over an intramedullary nail in patients with short stature or leg-length discrepancy: a comparative study by Guo et al. [14].

A search using the term ‘tibia’ performed on the Cochrane Database of Systematic Reviews revealed no studies suitable for review. References included in each of the selected papers were also examined in order to identify possible suitable studies but none were found. The literature search was performed independently by both authors, and disagreements regarding suitability for inclusion were resolved by discussion.

Methods of data analysis were specified through preliminary discussion and documented using a modification of the Cochrane Review Group’s data extraction template. This was performed initially by the primary author (SJ) and checked by the senior author (PH) for omissions. Specifically, data were extracted from each study with regard to number of participants in each study group, length of follow-up and outcome measures (external fixation index, consolidation index and complications, that is, pin site infections, deep infections, need for further surgical procedure). Both a difference in the mean and absolute values were used as summary measures for analysis of the results of the studies.

## Results

Six papers were identified as suitable for this review, and their results summarised and presented in Table 1. A summary of complications is given in Table 2 according to the system of Paley [3]. Due to the heterogeneity of methods used for measuring the external fixation and the healing or consolidation indices, we were unable to pool the data for meta-analysis. A critical appraisal of the methodology of each study is presented in chronological order of publication date including risk of bias and the potential effect of this on data interpretation.

**Table 1:** Characteristics and results of studies

Study	Design	Experimental group	Control group	Outcome measures^a^	Length of follow-up (mean, months)	Results in experimental group (experimental vs. control groups, mean)^†^
Watanabe et al. [9]	Retrospective case–control study	13 tibial lengthenings with external fixator and nail	17 tibial lengthenings with external fixator	Lengthening Distraction index External fixation index Consolidation (or healing) index Complications	Experimental: 48 Control: 42	Greater lengthening (6.8 vs. 5.0), lower external fixation index (18.0 vs. 41.2) and fewer complications (9 vs. 24) No difference in distraction (14.9 vs. 13.8) or consolidation (45.1 vs. 41.0) indices
Park et al. [10]	Retrospective case–control study	56 tibial lengthenings with external fixator and nail	32 tibial lengthenings with external fixator	Lengthening External fixation index Consolidation index Functional status Complications	Experimental: 40 Control: 48	Lower external fixation index (0.9 vs. 2.2) and fewer complications (69 vs. 82) No difference in lengthening (6.4 vs. 5.9) or consolidation index (1.7 vs. 2.1)
Popkov et al. [11]	Prospective comparative case–control study	20 tibial lengthenings with external fixator and nail	58 tibial lengthenings with external fixator	Lengthening Duration of osteosynthesis Consolidation index	Not stated	Lowest consolidation index in congenital group undergoing bifocal lengthening with a nail (16.3) Lowest consolidation index in acquired group undergoing monofocal lengthening with a nail (22.7) Greater lengthening and duration of osteosynthesis Complications incompletely reported
Sun et al. [12]	Retrospective case-matched series	49 tibial lengthenings with external fixator and nail	49 tibial lengthenings with external fixator	External fixation index Consolidation index Outcome score Complications	Experimental:23.6 Control: 25.1	Lower median external fixation index (1.1 vs. 1.3), consolidation index (1.5 vs. 1.8), higher outcome score (96 vs. 88) and fewer complications (3.0 vs. 3.7, per segment)
Sun et al. [13]	Retrospective case–control study	70 tibial lengthenings with external fixator and nail	56 tibial lengthenings with external fixator	External fixation index Consolidation index Complications	Not stated	Lower external fixation (1.1 vs. 1.7) and consolidation indices (1.5 vs. 1.8) More complications (210 vs. 190)
Guo et al. [14]	Retrospective case–control study	51 tibial lengthenings with external fixator and nail	23 tibial lengthenings with external fixator	Lengthening index External fixation index Consolidation index	Experimental:41 Control: 38	Lower external fixation index (17.4 vs. 40.0) and fewer mean number of complications per tibia (0.47 vs. 1.0) No difference in lengthening (13.3 vs. 14.4) or consolidation (40.7 vs. 40.6) indices

^a^Please refer to glossary for terms

^†^*p* < 0.05 denotes significance difference

**Table 2:** Complication reporting

Study	Experimental group	Complications	Infections	Further surgical procedure	Control group	Complications	Infections	Further surgical procedure
Watanabe et al. [9]	13 tibial lengthenings with external fixator and nail	Problems: 4 Obstacles: 5 Sequelae: 0 Total: 9	Pin Site: 1 Deep: 0	Contracture: 2 Nail fracture/protrusion: 2 Total: 4	17 tibial lengthenings with external fixator	Problems: 10 Obstacles: 9 Sequelae: 5 Total: 24	Pin Site: 10 Deep: 0	Contracture: 1 Delayed consolidation: 2 Axial deviation: 2 Refracture: 2 Total: 6
Park et al. [10]	56 tibial lengthenings with external fixator and nail	Problems: 35 Obstacles: 33 Sequelae: 1 Total: 69	Pin Site: 13 Deep: 0	Contracture: 3 Broken wire: 22 Premature consolidation: 5 Distal fibula migration: 2 Ankle valgus: 2 Total: 34	32 tibial lengthenings with external fixator	Problems: 19 Obstacles: 60 Sequelae: 3 Total: 82	Pin Site: 9 Deep: 0	Contracture: 5 Broken wire: 38 Axial deviation: 5 Delayed consolidation: 5 Premature consolidation: 5 Distal fibula migration: 2 Ankle valgus: 2 Total: 62
Popkov et al. [11]	20 tibial lengthenings with external fixator and nail	Incompletely reported	Incompletely reported	Delayed union: 1 Premature nail removal: 3 Total: 4	58 tibial lengthenings with external fixator	Incompletely reported	Incompletely reported	Axial deviation: 2 Fracture: 7 Pin site infection: 4 Pin tract osteomyelitis: 2 Total: 15
Sun et al. [12]	49 tibial lengthenings with external fixator and nail	Problems: 19 Obstacles: 103 Sequelae: 23 Total: 145	Pin Site: 13 Deep: 1	Incompletely reported	49 tibial lengthenings with external fixator	Problems: 39 Obstacles: 83 Sequelae: 58 Total: 180	Pin Site: 21 Deep: 0	Incompletely reported
Sun et al. [13]	70 tibial lengthenings with external fixator and nail	Problems: 39 Obstacles: 159 Sequelae: 12 Total: 210	Pin Site: 31 Deep: 5	Pin site infection: 7 Contracture: 56 Delayed consolidation: 24 Axial deviation: 11 Nail impingement: 1 Total: 100	56 tibial lengthenings with external fixator	Problems: 30 Obstacles: 105 Sequelae: 44 Total: 179	Pin Site: 20 Deep: 1	Contracture: 24 Delayed consolidation: 8 Axial deviation: 29 Premature consolidation: 1 Callus subsidence: 30 Total: 92
Guo et al. [14]	51 tibial lengthenings with external fixator and nail	Problems: 19 Obstacles: 5 Sequelae: 0 Total: 24	Pin Site: 8 Deep: 0	Incompletely reported	23 tibial lengthenings with external fixator	Problems: 20 Obstacles: 3 Sequelae: 0 Total: 23	Pin Site: 11 Deep: 0	Incompletely reported

### Watanabe et al. [9]

This retrospective case–control study compared lengthening, mean distraction index (DI), mean external fixation index (EFI), mean consolidation index (CI) and complications between 17 tibiae treated with an external fixation device (control group) and 13 tibiae treated with an external fixation device over a nail (experimental group). The results showed a significantly greater mean amount of lengthening, a significantly lower EFI and a fewer number of complications in the experimental group. There was no statistically significant difference between the groups in terms of DI and CI. There was one pin site infection in the experimental group and 10 in the control group. There were no cases of deep infection in either group. There were 4 further surgical interventions in the experimental group and 6 in the control group. Although the mean operating time in the experimental group was approximately 1 h longer, there was no significant difference in blood loss between the groups.

This study gave clear inclusion and exclusion criteria for patient selection, and there was an accurate description of the surgical techniques and long-term follow-up. Unfortunately, the study groups were poorly matched. This resulted in paediatric patients being excluded from the experimental group, whereas they were included in the control group. This is important as immature bone has different healing properties to mature bone and therefore adds an important confounding variable. There was also a change in the surgeon’s practice during the study in which use of the monolateral Orthofix frame was changed to the circular Ilizarov frame due to technical difficulties with the former. These patient sub-groups were not analysed separately. For the reported complication rates, statistical analysis was not performed. Although this study concluded that tibial lengthening over a nail is associated with a shorter external fixation time and fewer complications, its clinical relevance must be viewed with caution.

### Park et al. [10]

This was another retrospective case–control study involving a larger number of patients; it compared lengthening, mean EFI, mean CI and the number of complications between 32 tibiae lengthened with an Ilizarov frame and 56 tibiae lengthened with an Ilizarov frame over an intramedullary tibial nail. The results showed that there was a statistically significant shorter duration of time spent in frame and fewer complications in the group lengthened over a nail. There was no significant difference in the lengthening or healing index. There were 13 pin site infections in the experimental group and 9 in the control group. There were no cases of deep infection in either group. There were 34 further surgical interventions in the experimental group and 62 in the control group.

This well-structured study had a large sample size consisting of well matched patients. The same surgical technique and instrumentation was used in both groups, and this was described clearly. However, there was an important element of recruitment bias involved. As the public health system in South Korea would not fund tibial lengthening procedures over an intramedullary nail, some patients opted for the standard method of lengthening. Despite this, there were actually more patients recruited into the experimental arm of the study over the 8-year period. There was an added variable in the experimental group in that some patients required reaming of the medullary canal in order to fit the nail. This has been shown to affect endosteal blood supply which may subsequently affect healing or consolidation [15]. This variable was not further analysed and therefore contributes an element of performance bias. This study produced results similar to contemporaneous literature in terms of a lower external fixation index and fewer complications associated with tibial lengthening over an intramedullary nail.

### Popkov et al. [11]

This prospective non-randomised study compared the duration of external fixation and the CI in paediatric patients undergoing both upper and lower limb lengthening with an Ilizarov frame either with or without a flexible intramedullary nail. The sample population was further sub-classified into those with congenital or acquired causes of limb length discrepancy. The authors hypothesised that the CI would be reduced in those treated with a nail. Both monosegmental and bifocal lengthenings were undertaken for acquired and congenital causes of limb length discrepancy in a large number of cases. Their results showed that in the congenital aetiology cohort of tibial lengthening, there was a significantly lower CI in those that underwent bifocal distraction osteogenesis but not in those that underwent monofocal distraction osteogenesis. However, in the acquired aetiology cohort of tibial lengthening, the reverse was true. There was a trend towards a lower CI in those lengthened over a nail. The EFI was not calculated, and complications were incompletely reported. However, there were 4 further surgical interventions in the experimental group and 15 in the control group including 2 cases of pin track osteomyelitis.

Despite being the only prospective study in this review, the patients were not randomised and were given a choice whether to proceed with lengthening over a nail or with an external fixator alone. The authors came to similar conclusions in their paediatric population as others have in adult populations using rigid intramedullary nails. The small sample size may have been responsible for the inability to show a significant benefit consistently to lengthening over a flexible intramedullary nail across the aetiologies.

### Sun et al. [12]

This retrospective study matched patients based on the amount of lengthening, percentage lengthening, patient age and difficulty of the procedure. This was done in order to limit the confounding variables seen in previous studies. The case-matched groups involved 49 tibiae which underwent lengthening in a hybrid Ilizarov fixator compared to 49 tibiae which underwent lengthening over an intramedullary unreamed tibial nail. Their results showed that there was a significantly lower mean EFI, a lower mean CI, a higher outcome score and fewer complications in the group whose tibiae were lengthened over an intramedullary nail. There were 13 pin site infections in the experimental group and 21 in the control group. There was one deep infection in the group lengthened over a nail, and this was due to local infection at the corticotomy site. There was a very high complication rate compared to other studies. The authors attributed this to routine prophylactic nerve release in all patients which they considered an obstacle. The incidence of further surgical intervention was not clearly reported.

The same surgeon performed all the operations and used the same equipment; the surgical technique was described clearly and reproducible. The analyses for comparison of all the numerical data were presented via a combination of appropriate statistical tests. Unfortunately, patients whose lengthening percentages were of less than 5 % were recruited. This excludes a significant number of clinically relevant patients undergoing greater lengthening procedures and is important. Another methodological error was in the group lengthened over a nail where the healing point was when there was radiographic evidence of two healed cortices, whereas in the control group, this was taken to be at the point of three healed cortices, a point likely to occur later in the consolidation process. This clearly has the potential for influencing the CI and adds a significant amount of detection bias. Conclusions were again similar to other comparative studies but flaws in patient recruitment, outcome reporting and the high complication rate must be considered.

### Sun et al. [13]

The same authors presented another study comparing 70 tibiae which underwent lengthening with an Ilizarov hybrid fixator over an intramedullary nail to 56 tibiae which underwent lengthening with the external fixator alone. The primary outcome measure in this study was callous progression as measured by the pixel value ratio (PVR) and through the use of the Ru Li classification [16, 17]. Secondary outcome measures were mean lengthening, mean EFI, mean CI and mean duration of external fixation. The CI was defined in terms of the PVR so cannot be accurately compared to previous studies. The results showed that there was a significantly lower PVR (i.e., more mature callous) and significantly more homogenous callous progression in nail group. Whilst the results also stated that there was a lower EFI and CI but longer duration of external fixation in this group, statistical analysis was not applied to these particular outcome measures. There was a greater mean incidence of complication per lengthening segment in the control group but statistical analysis was not performed. There were 31 pin site infections in the experimental group and 20 in the control group. There were 5 cases of deep infection in the experimental group requiring removal of the nail and systemic antibiotic therapy. There was one deep infection in the control group which required debridement and antibiotic therapy. There were 100 further surgical interventions in the experimental group and 92 in the control group. Again, the authors attributed their high complication rate to routine prophylactic nerve release but the high rate of reoperation in both groups remains a concern and may also be due to surgical experience or technique.

Utilising the largest sample size to date, this study had a clear hypothesis that callus progression could be altered through lengthening over an intramedullary nail and they were able to prove this in their sample group. A major limitation of this study is that the treatment groups were poorly matched for aetiology. This may have an impact as there is a recognised risk that in the achondroplastic population largely prevalent in the control group, callus maturity is slower and this may have influenced the results. These patients also required bifocal osteotomies which may also have affected the rate of healing. A similar concern arises to that of the previous study in that the recognised point of cortical healing was different in both groups, that is, two or three united cortices for the group lengthened over a nail and the group lengthened in the standard manner, respectively. The primary aim of this review was to compare the duration of external fixation and rate of healing in patients lengthened with or without an intramedullary nail. Whilst the results of this study suggest that these factors are positively influenced by lengthening over an intramedullary nail, statistical analysis was not performed.

### Guo et al. [14]

This retrospective case–control study compared 23 tibiae lengthened with an Ilizarov external fixator and 51 tibiae lengthened with an Ilizarov external fixator over an intramedullary nail. The results stated that there was a significantly lower mean EFI and fewer complications per tibia in the group lengthened over an intramedullary nail, but no significant difference in mean lengthening or consolidation indices. There were 8 pin site infections in the experimental group and 11 in the control group which was a statistically significant difference. There were no cases of deep infection in either group. The rate of further surgical intervention was not clearly reported.

This study had clear inclusion and exclusion criteria, ethical approval and good recruitment numbers, but there was no evidence of a power study for accurate statistical analysis. The same surgeon performed all the operations and used the same equipment consistently according to the clearly documented and reproducible surgical technique. The major limitations of this study were the lack of homogeneity between the groups in terms of aetiology. As before, economic issues prevented some patients from being treated with an intramedullary nail which added some selection bias. Nevertheless, similar conclusions to other studies were reached in that shorter times were need for external fixation with fewer complications in the group lengthened over a nail.

## Discussion

The Ilizarov method for distraction osteogenesis is a well-established and widely used technique in limb lengthening. Tibial lengthening with the use of an external fixator takes many months and has recognised complications such as infection, joint contracture and deformity. In lengthening over an intramedullary nail, it is hypothesised that the overall time spent in an external fixator can be reduced and therefore improves patient satisfaction and reduce complications.

This review has highlighted six studies, investigating these key points. Generally, these studies had notable methodological flaws such as their retrospective nature, selection discrepancies in allocation of the patients to treatment groups and had mixed quality of reporting complications and reoperation rates. There was also a lack of homogeneity in aetiology between the patient groups in all but three of the studies.

In analysing the results of these studies, it is important to scrutinise the outcome measures in terms of clinical relevance. The main reported outcome measures were the EFI and the CI. Indices are used rather than absolute values as it is important to relate the duration of lengthening or consolidation to the actual amount of lengthening. All the studies showed that the EFI was significantly lower in the group lengthened over an intramedullary nail except for the second study by Sun et al. where statistical analysis was not performed. In practice, this means that in this group, the external fixator was removed at an earlier time, that is, when the desired lengthening had been achieved. This is in keeping with the recommended surgical technique [18]. In the group treated with the standard method of lengthening, the external fixator was removed when there radiographic evidence of cortical union. This point at which the external fixator was removed is therefore measured on different terms and cannot be compared directly as it is likely that radiographic union occurs later than when final lengthening is achieved. However, this is important clinically as it means that patients lengthened with an intramedullary nail will have spent less time in an external fixator. This observation was seen consistently in the reviewed studies.

It may seem more appropriate to consider the CI. This is a measure of time taken for actual consolidation of the regenerate in relation to the actual amount of lengthening. Clinically, this is relevant as it indicates when the patient can begin to fully weight-bear. Only two of the studies showed a difference in CI between the groups, both by Sun et al. Their first study used different radiographic parameters for each group to assess healing, and their second study did not perform a statistical analysis to their results. Therefore, it cannot be concluded that the rate of consolidation is altered by tibial lengthening over an intramedullary nail.

The reporting of complications was of mixed quality, especially in terms of patients requiring further surgical procedures. Three of the studies reported complications inadequately, and conclusions were difficult to draw. It would have been more useful to have reported the number of complications per tibia which could then be directly compared between the groups. Whilst pin site complication reporting was good, no specific details were provided on the actual regimes of pin site care. Overall, there was no difference between the numbers of pin site infections seen in the studies. In general, there was a trend for the control group requiring more secondary procedures than the group lengthened over a nail, but there were more deep infections seen with the latter. These required surgical debridement and long-term antibiotic therapy. The long-term results of these patients were not presented.

A limitation of this review was an inability to synthesise the results in terms of meta-analysis. Due to the different techniques used to measure the results, particularly the CI, it was not deemed accurate enough to pool the results. Also, retrieval of some data was incomplete because some studies failed to report length of follow-up or complication rates. We decided to include these studies as they were able to provide data for another outcome measure.

## Conclusion

Patients whose tibiae are lengthened with the Ilizarov method over an intramedullary nail spend less time in an external fixator as compared to those who are lengthened in the conventional manner. This has obvious implications in terms of patient comfort and satisfaction. There is no reliable evidence to suggest that the rate of consolidation or occurrence of complication is any difference between the two groups. In order to provide further answers to these questions, prospective randomised clinical trials involving homogeneous patient groups are required.

## Glossary of terms

Distraction index: duration of lengthening (days) divided by length gained (cm)
External fixation index: duration of external fixation (days) divided by length gained (cm)
Consolidation or healing index: duration of consolidation (measured from application of external fixation to radiographic consolidation of regenerated bone in days) divided by length gained (cm)
Lengthening index: duration of distraction phase divided by length gained (cm)
Percentage increase: length gained (cm) divided by original length (cm)
Pixel value ratio: (pixel value of proximal host bone + pixel value of distal host bone)/2/pixel value of regenerate
Total lengthening: total amount of length (cm) gained after removal of external fixator
